# Metabolic Acidosis Increases Intracellular Calcium in Bone Cells Through Activation of the Proton Receptor OGR1

**DOI:** 10.1359/jbmr.081015

**Published:** 2008-10-13

**Authors:** Kevin K Frick, Nancy S Krieger, Keith Nehrke, David A Bushinsky

**Affiliations:** Division of Nephrology, Department of Medicine, University of Rochester School of Medicine and Dentistry Rochester, New York, USA

**Keywords:** bone, acidosis, proton, intracellular calcium, bone resorption, osteoblast

## Abstract

Metabolic acidosis increases urine Ca without increasing intestinal absorption, leading to bone Ca loss. It is unclear how bone cells detect the increase in proton concentration. To determine which G protein-coupled proton sensing receptors are expressed in bone, PCR was performed, and products were detected for *OGR1*, *TDAG8*, *G2A*, and *GPR4*. We tested the hypothesis that the G protein-coupled proton sensor, OGR1, is an H^+^-sensing receptor in bone. To determine whether acid-induced bone resorption involves OGR1, we incubated mouse calvariae in neutral pH (NTL) or acidic (MET) medium ± the OGR1 inhibitor CuCl_2_. CuCl_2_ decreased MET-induced Ca efflux. We used fluorescent imaging of perfused bone cells to determine whether MET increases Ca_i_. Perfusion with MET induced a rapid, flow-independent, increase in Ca_i_ in individual bone cells. To determine whether transfection of *OGR1* into a heterologous cell type would increase Ca_i_ in response to H^+^, we perfused Chinese hamster ovary (CHO) cells transfected with mouse *OGR1* cDNA. Perfusion with MET induced a rapid increase in Ca_i_ in *OGR1*-transfected CHO cells. These data indicate that OGR1 induces an increase in Ca_i_ in response to MET and is a prime candidate for an osteoblast proton sensor.

## INTRODUCTION

Chronic metabolic acidosis alters calcium (Ca) homeostasis by increasing urine Ca excretion without a corresponding increase in intestinal Ca absorption, leading to depletion of the primary Ca reservoir in the body, the skeleton.(1,2) In vivo, mild metabolic acidosis, induced by ample dietary protein intake and an age-related decline in acid excretion, may deplete bone Ca stores.(1–3) This process can be modeled in vitro by culturing neonatal mouse bone in physiologically acidic medium.(4) We have found that, in the earliest time periods (≤24 h), bone calcium efflux is directly related to the magnitude of the fall in pH and caused by non-cell-mediated physicochemical mineral dissolution.(5,6) In contrast, at later time periods (>24 h), the continued calcium efflux is caused by cell-mediated bone resorption.(7–10) Metabolic acidosis regulates both osteoblastic and osteoclastic activity. Acidosis decreases expression of osteoblastic extracellular matrix proteins, including type 1 collagen,(7,8) osteopontin, and matrix gla protein,(11,12) and increases expression of COX-2(10) and RANKL.(9,13) Acidosis also increases secretion of osteoclastic β-glucuronidase.(8)

How the bone cells sense the changes in extracellular pH (proton concentration, [H^+^]) is not clear. Various families of acid-sensitive ion channels including the ASICs, TRPV1, and TASK are known to be expressed in pain sensing,(14) skeletal muscle,(15) and taste cells.(16,17) In addition, there is a family of G protein-coupled proton-sensing receptors.(18) This small family (40–50% shared homology) of G protein-coupled receptors includes the ovarian cancer G protein-coupled receptor 1, OGR1 (also called GPR68),(19) GPR4,(19) T-cell death-associated gene 8 (TDAG8, also called GPR65),(20,21) and G2A (also called GPR132).(21,22) These receptors are coupled either to phosphoinositide metabolism and increased Ca_i_ (OGR1 and G2A)(19,22) or alteration in adenylate cyclase activity (GPR4 and TDAG8).(19–21) Ludwig et al.(19) reported that OGR1 is expressed in mouse osteoblasts and that reduction in pH (increases in [H^+^]) leads to an accumulation of phosphoinositide metabolites, making OGR1 a prime candidate for an osteoblastic proton sensor.

To test the hypothesis that OGR1 acts as an H^+^-sensing receptor in bone cells, we studied whether an inhibitor of OGR1 (CuCl_2_) would diminish acidosis-induced Ca efflux from bone, whether metabolic acidosis would increase Ca_i_ in cultured bone cells, and whether transfection of *OGR1* into a heterologous cell type would permit cells to mimic the Ca_i_ response to acidosis of primary bone cells. We found that CuCl_2_ inhibits the acidosis-induced increase in Ca efflux from bone cells and that primary murine calvarial bone cells respond to a decrease in extracellular pH with an increase in Ca_i_. We found that Chinese hamster ovary fibroblasts (CHO cells) transfected with murine *OGR1* cDNA now respond to acidosis with increased Ca_i_. These data are consistent with a primary role for OGR1 as a sensor for H^+^ in bone cells.

## MATERIALS AND METHODS

### Reagents

All reagents, unless otherwise specified, were obtained from Sigma-Aldrich (St Louis, MO, USA).

### Animals and cells

CD-1 mice were obtained from Charles River (Wilmington, MA, USA). CHO fibroblasts were a gift from Patricia Hinkle (University of Rochester, Rochester, NY, USA) and grown in αMEM (Invitrogen, Carlsbad, CA, USA) containing 5% heat-inactivated fetal bovine serum (FBS; Invitrogen) and penicillin (100 U/ml; Sigma-Aldrich).

### RNA isolation and PCR

RNA was isolated from primary calvarial cells using a Qiagen RNeasy kit (Valencia, CA, USA) as previously described.(9) Total RNA (1 μg) was reverse transcribed to first-strand cDNA using an iScript cDNA synthesis kit (Biorad, Hercules, CA, USA) and amplified by PCR using iQ SYBR-green in an iCycler thermocycler (Biorad).

PCR primers were synthesized by Integrated DNA Technologies (Coralville, IA, USA) and were as follows: *OGR1* (forward, 5′-CCTCAACCTGTTTCGGACGTGC; reverse, 5′-CCACATATCAGCTCTCCCCGTCTC); *G2A* (forward, 5′-GACATGGATGCCGTGTGTGCC; reverse, 5′-CCCAGACGGTGACTCAGAGGAC); *GPR4* (forward, 5′- ACCGAGCGCCAGGAGAAAGTC; reverse, 5′-GGAGGCACTGCCCAGACAGC); and *TDAG*8 (forward, 5′-CCTCAACCTGTTTCGGACGTGC; reverse, 5′-CCACATATCAGCTCTCCCCGTCTC). PCR products were resolved by electrophoresis in agarose (Invitrogen) and migration rate compared with a size standard (1 kb Plus DNA Ladder; Invitrogen).

### Effect of CuCl_2_ on bone calcium efflux

Calvariae from 4- to 6-day-old CD-1 mice were dissected and incubated in 2.8 ml DMEM (Lonza, Walkersville, MD, USA) containing 15% heat-inactivated horse serum (Invitrogen), heparin (10 USP units/ml; Baxter Healthcare, Deerfield, IL, USA), and penicillin (100 U/ml) in 35-mm dishes at neutral pH (NTL, pH ≍≈ 7.4) or at a physiologically acidic pH (MET, pH ≍≈ 7.1) produced by a primary reduction in [HCO_3_^−^], as a model of metabolic acidosis.(6,8,23) Calvariae were incubated in NTL or MET each without or with 100 μM CuCl_2_ for a total of 48 h. Immediately before adding two bones per dish on a square, 9-cm^2^ stainless steel mesh grid supported on 0.5-cm legs, 1 ml of medium was removed to determine preincubation pH, PCO_2_ and [Ca]. At the conclusion of the first 24-h incubation period, medium was removed, analyzed for pH, PCO_2_, and [Ca], and replaced with similar, fresh preincubated medium. At the conclusion of the second 24-h incubation period, medium was again analyzed for pH, PCO_2_, and [Ca]. Throughout all experiments, the PCO_2_ was maintained at the physiologic normal of ≍≈40 mmHg. To closely replicate physiological conditions, only the HCO_3_^−^/CO_2_ buffer system was used; no other buffers were added to the medium. All medium was preincubated at PCO_2_ ≍≈40 mmHg, 37°C, for at least 3 h before use. The number of pairs of bones chosen in each group was determined by power analysis using actual means and SD from initial experiments with an *n* = 4. We assumed probability of a type I error (α) = 0.05 and the probability of a type II error (β) = 0.10. We used Statistica (version 6.0; StatSoft, Tulsa, OK, USA). For a statistical power (1 − β) goal of 0.9, we needed eight pairs of bones in each group. In the protocols above, we used 10–12 pairs of bones in each group.

### Primary bone cell culture

Primary bone cells, which are almost exclusively osteoblasts,(24) were isolated from neonatal CD-1 mouse calvariae immediately after dissection as described previously.(24,25) Briefly, bones were washed in PBS containing 4 mM EDTA for 10 min at 37°C and incubated in HEPES (25 mM), pH = 7.4, containing 2 mg/ml collagenase (Wako Pure Chemicals, Dallas, TX, USA) and 90 μM Nα-tosyl-l-lysyl chloromethyl ketone for three sequential 20-min digestion periods at 37°C in a shaking water bath. At the end of each digestion, released cells were collected and resuspended in HEPES buffer containing 1 mM MgSO_4_, and all three digests were pooled in DMEM (Lonza, Walkersville, MD, USA) containing 15% heat-inactivated horse serum (Invitrogen), heparin (10 USP units/ml; Baxter), and penicillin (100 U/ml) for plating on 8-mm glass coverslips (Warner Instruments, Hamden, CT, USA). Coverslips were used for imaging 1–5 days after initial plating.

### Cell imaging

**FIG. 4:**
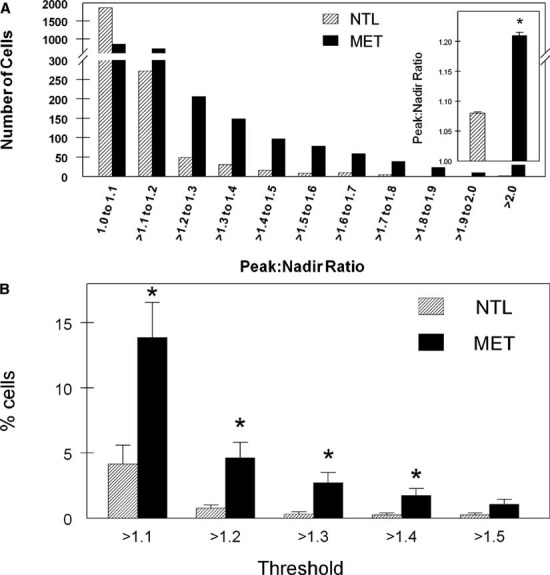
Quantitation of Ca_i_ in primary bone cells. Changes in Ca_i_ were quantified by two methods: (A) the distribution of cell number as a function of the peak to nadir ratio and (B) the percentage of cells with an increase in Ca_i_ above specific threshold values. (A) The ratio of the maximum to minimum Ca_i_ for each cell during the NTL and MET phases of perfusion was determined and the distribution of values plotted. The distributions were significantly different, *p* < 0.01. Inset, mean ± SE for all NTL and all MET cells. (B) The percentage of cells with an increase in Ca_i_ above each specified threshold. The peak in cell Ca_i_ was defined as an increase in Ca_i_ of greater than the specified threshold (1.1–1.5) over the mean of the previous five time points (time point taken every 2 s) for that cell. *n* = 2306 cells individually analyzed for A and B; *p < 0.05.

**FIG. 6:**
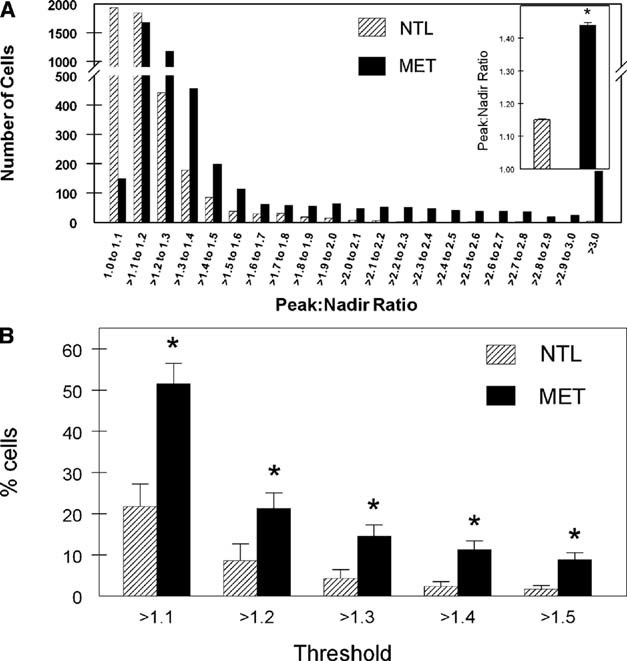
Quantitation of Ca_i_ in *OGR1*-transfected CHO cells. Changes in Ca_i_ were quantified by two methods: (A) the distribution of cell number as a function of the peak to nadir ratio and (B) the percentage of cells with an increase in Ca_i_ above specific threshold values. (A) The ratio of the maximum to minimum Ca_i_ for each cell during the NTL and MET phases of perfusion was determined and the distribution of values plotted. The distributions were significantly different, *p* < 0.01. Inset, mean ± SE for all NTL and all MET cells. (B) The percentage of cells with an increase in Ca_i_ above each specified threshold. The peak in cell Ca_i_ was defined as an increase in Ca_i_ of greater than the specified threshold (1.1–1.5) over the mean of the previous five time points (time point taken every 2 s) for that cell (*n* = 4758 cells individually analyzed for A and B; *p < 0.05).

To reduce background, DMEM without phenol red (Invitrogen) was used for perfusion while imaging. To closely replicate physiological conditions, only the HCO_3_^−^/CO_2_ buffer system was used; no other buffers were added to the medium. The acidity of the medium was adjusted by a primary reduction in [HCO_3_^−^] as a model of metabolic acidosis as previously reported.(6,8,23) The partial pressure of medium CO_2_ (PCO_2_) was maintained by gentle bubbling with 5% CO_2_ in air (AirGas, Rochester, NY, USA) for at least 15 min before perfusion. A continuous flow of equilibrated medium was established through an open microscope chamber (RC-26; Warner Instruments; ∼200-μl volume) by use of a peristaltic pump (∼1 ml/min) and aspiration. To reduce gas loss, where possible, Viton (DuPont, Wilmington, DE, USA) or fluorinated ethylene-propylene (FEP) tubing was used. Flow control was accomplished with a manually operated perfusion valve system (V-6; Warner Instruments). To load cells on coverslips with fura-PE3 (TefLabs, Austin, TX, USA), coverslips were washed with ice-cold PBS and incubated for 45 min at 37°C in DMEM containing 4.4 μM fura-PE3 and 48 μM pluronic acid F127 (Invitrogen). Coverslips were mounted in the chamber with flow of neutral medium. For stimulation with acidic medium, a continuous perfusion with neutral medium was switched at time = 0 to acidic medium. The chamber became acidified at ∼180 s as determined in a subset of experiments (*n* = 36), using a pH electrode in the chamber (Thermo, Beverly, MA, USA). Cells were visualized with a Nikon Eclipse 2000U inverted microscope (×20 objective; Nikon, Tokyo, Japan) equipped with a CCD camera (Cooke, Romulus, MI, USA). Each field was imaged every 2 s for 600 s. Intracellular Ca (Ca_i_) levels were measured from fluorescent emissions at 510 nm after excitation at 340 and 380 nm using a dual wavelength excitation system (TILL Photonics, Munich, Germany). Fluorescence data were interpreted and analyzed with TILLVision software (TILL Photonics). As calculations of absolute Ca_i_ require many assumptions and remain estimates, ratios of 340 nm/380 nm were used in this study and reported as relative fluorescence intensity.(26,27) The maximum peak to nadir ratio for each cell was recorded and used to construct the summary graphs (Figs. 4A and 6A).

### pH, PCO_2_, and total calcium measurements

Medium was sampled with a syringe, and pH and PCO_2_ were determined with a blood-gas analyzer (ABL5; Radiometer, Copenhagen, Denmark), and the concentration of medium bicarbonate ([HCO_3_^−^]) was calculated from pH and PCO_2_ as described previously.(6,28) Ca was measured by an ion selective electrode (Model 10; Nova Biomedical, Waltham, MA, USA).

### Stable transfection of CHO cells

A full-length cDNA for murine *OGR1* was recovered from calvarial cell RNA by standard techniques(29,30) and inserted into the mammalian expression vector pcDNA3.1/V5-His-TOPO (Invitrogen). The identity of the insert was confirmed by sequencing. Plasmid DNA was transfected into CHO cells with Lipofectamine LTX as directed by manufacturer (Invitrogen). Stable transfectants were selected for growth in medium containing 420 μg/ml G418 (Invitrogen). The V5 epitope tag was used to screen for m*OGR1* expression; transfectants selected for further study had ∼30% V5 + cells after staining with antiserum to V5 (Invitrogen).

### Statistical analyses

All values were expressed as mean ± SE. Tests of significance were calculated using Student's *t*-test, ANOVA with Bonferroni correction for multiple comparisons (means), or the Kolmogorov-Smirnov test (distributions) using conventional programs (Statistica; StatSoft, Tulsa, OK, USA) on a personal computer. *p* < 0.05 was considered significant.

## RESULTS

### Expression of H^+^-sensing receptors in bone cells

**FIG. 1:**
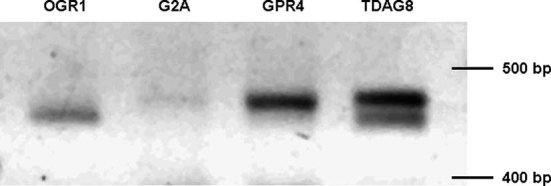
Expression of H^+^-sensing receptors in bone cells. RNA was isolated from primary calvarial cells, reverse transcribed, and amplified by PCR using primer sequences. PCR products were resolved by electrophoresis and migration rate compared with a size standard.

To determine the pattern of expression of the four known G protein-coupled proton sensing receptors (OGR1, G2A, GPR4, and TDAG8) in bone cells isolated from mouse calvariae, RNA was isolated and reverse transcribed and PCR was performed with specific primers (see the Materials and Methods section). We found that each of the four known proton receptors was present in the cultured bone cell RNA (Fig. 1). Whereas *G2A* expression appears less abundant than the other three isoforms, the image shows plateau levels, and absolute abundance was not determined.

### Effect of CuCl_2_ on bone resorption

**FIG. 2:**
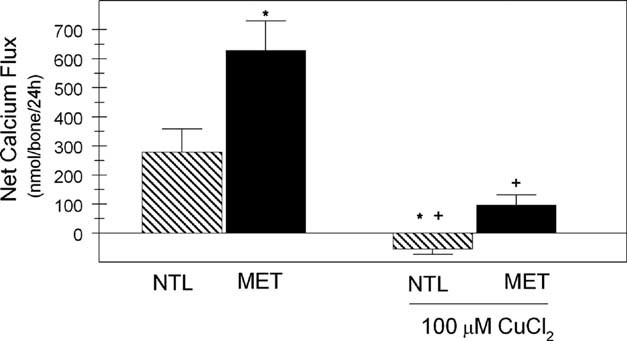
Effect of CuCl_2_ on bone Ca efflux. Calvariae from CD-1 mice were incubated in physiologically neutral (NTL) or acidic (MET) medium for 48 h in the absence or presence of 100 μM CuCl_2_ with a medium change at 24 h. Results are the mean ± SE for 10–12 pairs of calvariae in each group. *Different from NTL, *p* < 0.05; ^+^different from MET, *p* < 0.05.

To test the hypothesis that the initial step in acid-induced bone resorption involves signal transduction through OGR1, we incubated neonatal mouse calvariae in the absence or presence of the OGR1 inhibitor CuCl_2_ (100 μM).(19) CuCl_2_ has been shown to inhibit OGR1 through essential histidine residues.(19) Incubation of calvariae with physiologically acidic medium (MET, pH = 7.17 ± 0.02, PCO_2_ = 36.4 ± 0.9 mmHg, [HCO_3_^−^] = 12.9 ± 0.5 mM) led to a significant increase in net Ca efflux compared with incubation in neutral pH medium (NTL, pH = 7.45 ± 0.01, PCO_2_ = 35.8 ± 0.3 mmHg, [HCO_3_^−^] = 24.6 ± 0.2 mM; Fig. 2). Incubation of calvariae with physiologically neutral medium in the presence of CuCl_2_ (pH = 7.46 ± 0.02, PCO_2_ = 34.9 ± 1.1 mmHg, [HCO_3_^−^] = 24.4 ± 0.2 mM) led to a significant decrease in net Ca efflux compared with incubation in neutral pH medium in the absence of CuCl_2_. Incubation of calvariae with MET + CuCl_2_ (pH = 7.19 ± 0.03, PCO_2_ = 35.0 ± 0.8 mmHg, [HCO_3_^−^] = 12.9 ± 0.4 mM) significantly inhibited acid-induced Ca efflux and was not different from calvariae incubated in NTL + CuCl_2_ medium, consistent with the hypothesis that OGR1 activation is necessary for acid-induced bone Ca efflux.

### Measurement of Ca_i_ in primary bone cells

**FIG. 3:**
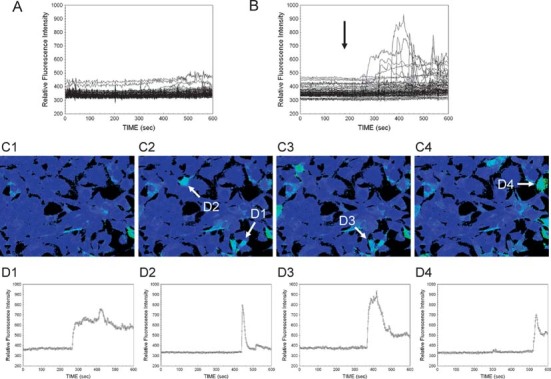
Imaging of Ca_i_ in primary bone cells. Primary bone cells on coverslips were loaded with fura-PE3 and perfused with physiologically neutral (NTL) or acidic (MET) medium. Ca_i_ levels were determined from fluorescent emissions at 510 nm after excitation at 340 and 380 nm using a dual wavelength excitation system. (A) Perfusion with NTL only. (B) Perfusion with NTL (0–180 s) followed by MET (182–600 s). Arrow, time of chamber acidification. Relative fluorescence as a function of time for all cells is shown in C (*n* = 79 individual cells). (C) Pseudocolor images of relative fluorescence intensity; C1, time = 0; C2, time = 198 s; C3, time = 221 s; C4, time = 288 s Arrows indicate selected cells whose change in fluorescence as a function of time is shown in D1–D4, respectively.

Because OGR1 has been shown to be coupled to phospholipase C, leading to increased IP_3_ production and subsequent increases in intracellular Ca (Ca_i_), we next determined whether Ca_i_ was altered in response to a physiologic reduction in pH. Changes in Ca_i_ were measured by loading primary bone cells with Fura-PE3 and measuring the ratio of fluorescent emissions at 510 nm after excitation at 340 and 380 nm. The bone cells were continuously perfused with neutral (NTL, pH = 7.40, PCO_2_ = 47 mmHg, [HCO_3_^−^] = 29 mM) medium, which did not lead to a significant alteration of Ca_i_ (Fig. 3A).

Cells were perfused with NTL followed by MET. Again, perfusion of the cells with NTL (pH = 7.42 ± 0.02, PCO_2_ = 44.4 ± 1.2 mmHg, [HCO_3_^−^] = 28.2 ± 1.0 mM) did not change Ca_i_ (Fig. 3B, 0–180 s). In contrast, when the cells were perfused with MET (pH = 6.90 ± 0.03, PCO_2_ = 41.8 ± 1.7 mmHg, [HCO_3_^−^] = 8.2 ± 0.8 mM), there was a substantial, rapid increase in Ca_i_ in multiple cells (Fig. 3B, 182–600 s). The increase in Ca_i_ in response to MET was asynchronous, occurred in individual cells at various times after the initial exposure to MET, and varied in magnitude and duration. A pseudocolor representation of selected fields among those analyzed for Fig. 3B is shown in Fig. 3C. The first frame (C1) indicates the initial time (time = 0 s), and the latter three frames show representative responses to acidification at 198, 221, and 288 s for C2, C3, and C4, respectively. Arrows indicate cells whose relative fluorescence intensity is shown in the corresponding traces in D1–D4. Cells shown in C1–C4 and corresponding traces D1–D4 show various patterns of response, with some cells displaying single peaks, whereas others exhibit more complex responses.

Changes in Ca_i_ were quantified by two methods: the distribution of cell number by the peak to nadir ratio and the percentage of cells with an increase in Ca_i_ above specific threshold values.

The ratio of the maximum to minimum Ca_i_ for each cell during the initial NTL and MET phases of perfusion was determined for all 300 time points for each cell and the distribution of values plotted. The distribution of the peak to nadir ratio for cells during the MET phase of perfusion was significantly different than that for the NTL phase of perfusion with more cells in MET having a greater peak to nadir ratio (*n* = 2306 cells in each group; Fig. 4A) and the mean value of the ratio (inset) was significantly increased with MET.

The percentage of cells with an increase in Ca_i_ above a specific threshold (1.1) was determined. A peak in cell Ca_i_ was defined as a >1.1 increase in area under the curve of Ca_i_ for each 2-s time interval compared with the mean area under the curve for the previous five time intervals for that cell. This calculation was repeated for threshold values of 1.2, 1.3, 1.4, and 1.5. Compared with the initial NTL phase of imaging, there was a significant increase in the percentage of cells with Ca_i_ peaks >1.1, >1.2, >1.3, and >1.4 during the MET phase of imaging (*n* = 2306 cells individually analyzed; Fig. 4B).

### Measurement of Ca_i_ in OGR1-transfected CHO cells

We next tested the hypothesis that expression of *OGR1* in CHO cells would lead to a MET-induced increase in Ca_i_. Nontransfected CHO cells do not increase Ca_i_ in response to MET (pH = 7.02, PCO_2_ = 52 mmHg, [HCO_3_^−^] = 13 mM) compared with NTL (pH = 7.37, PCO_2_ = 51 mmHg, [HCO_3_^−^] = 29 mM; *n* = 1234 cells in each group, % cells with peaks >1.2: NTL = 4.1 ± 2 versus MET = 2.7 ± 0.7, *p* = not significant; data not shown).

CHO cells were transfected with a plasmid encoding full-length mouse *OGR1* cDNA and selected for antibiotic resistance. Stable lines were screened for expression of a V5 epitope encoded by the expression plasmid; up to ∼30% of cells were positive for V5 expression in various lines, and the three maximally expressing lines were chosen for further study. The responses of these three lines were not different, and data for these lines were combined.

To determine whether Ca_i_ was altered in response to a physiologic reduction in pH, *OGR1*-transfected CHO cells were loaded with fura-PE3, and Ca_i_ was determined during continuous perfusion of NTL followed by MET medium.

**FIG. 5:**
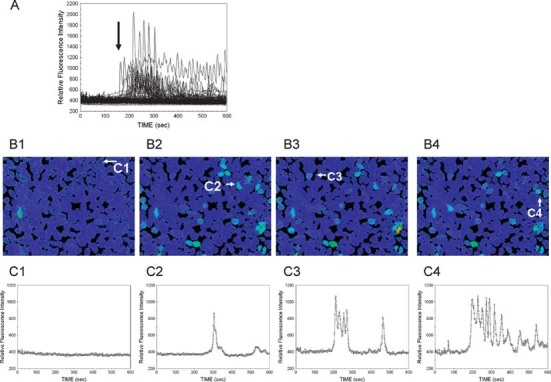
Imaging of Ca_i_ in *OGR1*-transfected CHO cells. CHO cells transfected with mouse *OGR1* on coverslips were loaded with fura-PE3 and perfused with physiologically neutral (NTL) or acid (MET) medium. Ca_i_ levels were determined from fluorescent emissions at 510 nm after excitation at 340 and 380 nm using a dual wavelength excitation system. (A) Perfusion with NTL (0–180 s) followed by MET (182–600 s). Arrow, time of chamber acidification. Relative fluorescence as a function of time for all cells is shown in B (*n* = 255 individual cells). (B) Pseudocolor images of relative fluorescence intensity; B1, time = 0; B2, time = 250 s; B3, time = 280 s; B4, time = 300 s Arrows indicate selected cells whose change in fluorescence as a function of time is shown in C1–C4, respectively.

The initial NTL (pH = 7.37 ± 0.02, PCO_2_ = 46.6 ± 0.7 mmHg, [HCO_3_^−^] = 26.6 ± 1.6 mM) phase of perfusion again did not lead to alteration of Ca_i_ (Fig. 5A, 0–180 s). Perfusion with MET (pH = 6.90 ± 0.03, PCO_2_ = 43.2 ± 1.3 mmHg, [HCO_3_^−^] = 8.2 ± 1.0 mM) led to a rapid increase in Ca_i_ in multiple cells (Fig. 5A, 182–600 s). The increase in Ca_i_ in response to MET was again asynchronous; the increase in Ca_i_ in individual cells occurred at various times after the initial exposure to MET and varied in magnitude and duration. Pseudocolor representations of selected fields among those analyzed for Fig. 5A are shown in Fig. 5B. The first frame (B1) indicates the initial time and the latter three frames show representative responses to acidification at 250, 280, and 300 s for B2, B3, and B4, respectively. Arrows indicate cells whose relative fluorescence intensity are shown in the corresponding traces in C1–C4. The cell indicated in B1 and C1 did not respond to acid. Cells shown in B2–B4 and corresponding traces C2–C4 illustrate various patterns of response, with some cells displaying single peaks, whereas others exhibit more complex responses. The period of time before changes in Ca_i_ were observed after MET reached the perfusion chamber was shorter than with primary bone cells (compare time to initial peaks in Fig. 5A with Fig. 3B).

Again, changes in Ca_i_ were quantified by two methods: the distribution of cell number by the peak to nadir ratio and the percentage of cells with an increase in Ca_i_ above specific threshold values.

The ratio of the maximum to minimum Ca_i_ for each cell during the initial NTL and MET phases of perfusion was determined for all 300 time points for each cell and the distribution of values plotted. Again the distribution of the peak to nadir ratio for cells during the MET phase of perfusion was significantly different than that for the NTL phase of perfusion with more cells in MET having a greater peak to nadir ratio (*n* = 4758 cells in each group; Fig. 6A) and the mean value of the ratio (inset) was significantly increased with MET.

The percentage of cells with an increase in Ca_i_ above a specific threshold (1.1) was determined. A peak in cell Ca_i_ was again defined as a >1.1 increase in area under the curve of Ca_i_ for each time point compared with the mean area under the curve for the previous five time points (time point taken every 2 s) for that cell. This calculation was repeated for threshold values of 1.2, 1.3, 1.4, and 1.5. Compared with the initial NTL phase of imaging, there was a significant increase in the percentage of cells with Ca_i_ peaks >1.1, >1.2, >1.3, >1.4, and >1.5 in the MET phase of imaging (*n* = 4758 cells individually analyzed; Fig. 6B).

## DISCUSSION

An acidic extracellular pH increases osteoclastic bone resorption through an increase in osteoblastic COX-2,(10) leading to an increase in RANKL(9,13) and subsequent increase in net Ca efflux from bone.(7–10) A class of H^+^-sensing G protein-coupled receptors, whose activity is mediated by release of the second messengers Ca_i_ or cAMP, has now been identified.(18) Stimulation of these H^+^ receptors is an attractive mechanism by which bone cells could sense and initiate the response to acidosis. In this study, we confirmed the expression of each of four known proton receptors OGR1, TDAG8, GPR4, and G2A by RT-PCR of RNA from primary bone cells (Fig. 1).

Because CuCl_2_ binds to essential histidine residues in OGR1,(19) we next showed that this compound significantly decreased acid-induced bone net Ca efflux, a marker of bone resorption, in cultured neonatal mouse calvariae. To our knowledge, the specificity of CuCl_2_ for proton receptors other than OGR1 has not be reported. The four histidine residues in OGR1 determined by Ludwig et al.(19) to be essential for acid induction of inositol phosphate formation are conserved in GPR4. On the basis of primary structure, the positioning of histidine residues in TDAG8 and G2A differs from OGR1. These structural features suggest that GPR4 might also be sensitive to CuCl_2_, whereas TDAG8 and G2A would be less susceptible; however, such predictions would require experimental testing.

The observed suppression of Ca release by CuCl_2_ in neutral conditions may indicate that OGR1 or similar receptors also have a role in establishing the basal rate of resorption. OGR1 has been previously detected in osteoblasts(19) and is coupled to phospholipase C and increases in diacylglycerol and inositol phosphate (IP) metabolism, leading to an increase in Ca_i_.(19,31) We found that a subset of cultured primary calvarial cells respond to acidosis pH with an increase in Ca_i_. To further support OGR1 as a prime candidate as an osteoblastic H^+^ sensor, we asked whether heterologous cells transfected with OGR1 would mount a similar Ca_i_ response to acidosis as do primary bone cells. CHO cells were stably transfected with mouse *OGR1* cDNA. When cultures of these transfectants were perfused with acidic medium, there was a prompt increase in Ca_i_ that was not present in nontransfected CHO cells. These data indicate that OGR1 is capable of transducing an increase in Ca_i_ in response to MET and is a prime candidate as the osteoblast proton sensor.

To replicate, to the extent possible, physiologic conditions, in this study, only the HCO_3_^−^/CO_2_ buffer system was used to adjust [H^+^]; medium pH was adjusted by varying the [HCO_3_^−^] while maintaining a constant physiologic PCO_2_. The open chamber used in the perfusion experiments permits adequate fluid flow while limiting fluid shear stress, which has been shown to alter Ca_i_.(32–34) We did not observe significant changes in Ca_i_ during perfusion with neutral pH medium, indicating that there was no response to either flow or the neutral pH medium (Fig. 3A).

With primary bone cells, the time from when the cells were acidified to their maximal increase in Ca_i_ and the magnitude of that increase were heterogenous among the cells. Not every cell in the field showed a measurable increase in Ca_i_ to acidosis. By visual examination, the responding cells were morphologically indistinguishable from cells that did not respond. It is not clear why some cells responded to an increase in [H^+^] and others did not. Whereas the primary bone cells used in this study are almost exclusively osteoblasts,(24) they are clearly a heterogenous population at different levels of maturation.(35–37) The ability of the cells to respond to acidosis with increased Ca_i_ may be related to their level of maturation. While the use of more acidic medium (greater [H^+^]) might have elicited a more robust response, we chose to use a perfusion pH within the pathophysiologic range (i.e., conditions that are compatible with life).(38–40) With the *OGR1*-transfected CHO cells, cell-to-cell variations in the Ca_i_ response to H^+^ may be the result of differences in expression of *OGR1*.

This study does not exclude that other G protein-coupled H^+^ sensors are involved in the response of bone to acidosis. The function of G2A as an H^+^ receptor is controversial and may be related to specific cell type,(30) although its activation would also lead to an increase in Ca_i_. However, there seemed to be little expression of G2A in cultured bone cells (Fig. 1). In contrast, although apparently abundant in cultured bone cells, TDAG8 and GPR4 are coupled to adenylate cyclase and should not directly alter Ca_i_.(19–21) Further studies will be needed to elucidate the potential roles, if any, of other proton receptors such as TDAG8 and GPR4, whose effect is mediated through cAMP in the response of bone to acid.

Other proteins that modulate biological responses to changes in extracellular [H^+^] include the pH-sensitive leak K^+^ channel TASK,(16,17) the vanilloid receptor TRPV1,(15) and the proton-gated, voltage-insensitive sodium channels, the ASICs.(14) Expression of *TASK* and *TRPV1* by osteoblasts has not been reported, although osteoclasts express *TRPV1*.(41) Human pre-osteoblasts express *ASIC* 1–4 and retain expression during mineralization.(42) Whereas the ASICs are responsive to pH increments in the range of 0.05–0.06 units,(43) their pH of half-maximal activation ranges from 6.5 to 4.4.(44) In the experiments reported here, pH was reduced from 7.4 to 6.9, well above the region of maximal responsiveness. However, with ASIC3, significant changes in current can be induced by reducing the bath pH from 8.0 to 7.0,(45) and it is possible that the pH range of the ASICs could be modulated by accessory proteins. Thus, we cannot exclude a role for ASICs in acid-induced bone resorption.

In this study we chose to focus on acid-induced changes in Ca_i_. In addition to its effects on inositol phosphate and Ca_i_, OGR1 in human aortic smooth muscle cells(46) or expressed in CHO or COS7 cells(31) also activates cAMP formation. However, the response to H^+^, as a function of the magnitude of the acidosis, is quite different for cAMP. Whereas IP accumulation is half-maximal at pH ∼ 7.1, the cAMP effect is half-maximal at pH ∼ 6.6, which is well outside the pH range generally thought to be compatible with life. We cannot, without further study, exclude a small contribution of increased cAMP to the OGR1-mediated increase in Ca_i_ shown in this study to acid-induced bone resorption. We have never found any measurable change in cAMP in response to MET in either intact calvariae or primary calvarial cells (unpublished data). A recent publication has studied acid induction of COX-2 in the human osteoblastic cell line NHOst.(47) These cells express *OGR1* as the sole G protein-coupled proton-sensing receptor. A reduction of the incubation pH to 6.7 caused a substantial increase in Ca_i_, which was sensitive to the Gα_q/11_ inhibitor YM-254890. Acid induction of Ca_i_ was also inhibited by an siRNA directed against OGR1.

OGR1 may have roles in multiple bone cell types. The primary cells used in this study are almost exclusively osteoblasts, and our results support the role of OGR1 in the osteoblastic response to acidosis. However, the importance of OGR1 in osteoclastogenesis has also been established. A decrease in medium pH from 7.4 to 7.0 induces Ca_i_ transients in rat osteoclasts, and these transients are suppressed by the phospholipase C inhibitor U73122.(48) In its proliferative state, the monocytic cell line RAW264.7 expresses low levels of OGR1 and acidification causes only a minor change in Ca_i_. Osteoclastic differentiation of RAW264.7 with RANKL increases OGR1 levels and the Ca_i_ response to acidification. A short hairpin RNA construct directed against OGR1 decreased the Ca_i_ response to acidification in transfected RAW264.7 cells.(49) Whereas acidification of osteoclastic cells stimulates NFATc1 translocation to the nucleus,(48) acidification also promotes osteoclast survival through a Ca- and ERK-dependent pathway that is NFAT independent.(49)

The expression of mouse *OGR1* in CHO cells models the acid-induced increase in Ca_i_ in primary bone cells, suggesting that OGR1 acts as the primary mediator of the response of osteoblasts to increased proton concentration. The data presented in this study and that of Tomura et al.(47) are consistent with a model in which proton activation of OGR1 leads to increased Ca_i_ levels. This increase acts as a second messenger to mediate the effects of acidosis on osteoblasts, including increased COX-2(10) and RANKL(9,13) expression, which result in increased osteoclastic bone resorption.
